# Use of vasopressors in patients with acute kidney injury on continuous kidney replacement therapy

**DOI:** 10.1371/journal.pone.0315643

**Published:** 2024-12-19

**Authors:** Ambika Ramesh, Akshith Doddi, Aisha Abbasi, Mohammad A. Al-Mamun, Ankit Sakhuja, Khaled Shawwa

**Affiliations:** 1 Department of Medicine, West Virginia University, Morgantown, West Virginia, United States of America; 2 Department of Pharmaceutical Systems and Policy, West Virginia University, Morgantown, West Virginia, United States of America; 3 Division of Data-Driven and Digital Medicine, Department of Medicine, Icahn School of Medicine at Mount Sinai, New York, New York, United States of America; 4 Division of Nephrology, Department of Medicine, West Virginia University, Morgantown, West Virginia, United States of America; Mayo Clinic College of Medicine and Science, UNITED STATES OF AMERICA

## Abstract

**Objective:**

To investigate whether the use of a specific vasopressor was associated with increased mortality or adverse outcomes in patients with acute kidney injury (AKI) receiving continuous kidney replacement therapy (CKRT).

**Methods:**

Patients with AKI who underwent CKRT between 1/1/2012-1/1/2021 at a tertiary academic hospital were included. Cox proportional hazard model was used to assess the relationship between time-dependent vasopressor dose and in-hospital mortality.

**Results:**

There were 641 patients with AKI that required CKRT. In-hospital mortality occurred in 318 (49.6%) patients. Those who died were older (63 vs 57 years), had higher SOFA score (10.6 vs 9) and lactate (6 vs 3.3 mmol/L). In multivariable model, increasing doses of norepinephrine [HR 4.4 (95% CI: 2.3–7, p<0.001)] per 0.02 mcg/min/kg and vasopressin [HR 2.6 (95% CI: 1.9–3.2, p = 0.01)] per 0.02 unit/min during CKRT were associated with in-hospital mortality. The model was adjusted for vasopressor doses and fluid balance, SOFA score, lactate and other markers of severity of illness. Baseline vasopressor doses were not associated with mortality. Most vasopressors were associated with positive daily fluid balance. Among survivors at day 30, mean values of vasopressors were not associated with persistent kidney dysfunction.

**Conclusion:**

The associations between norepinephrine and vasopressin with in-hospital mortality could be related to their common use in this cohort.

## Introduction

Patients with acute kidney injury (AKI) who receive continuous kidney replacement therapy (CKRT) in the intensive care unit (ICU) often have unfavorable outcomes. Patients who require CKRT are often on vasopressors. Studies evaluating the use of vasopressor during CKRT are limited in terms of size and patient demographics. It is unknown whether the pharmacokinetics and pharmacodynamics are affected. In one study, vasopressin was found to have a sieving coefficient of 1 in pediatric patients undergoing continuous veno-venous hemofiltration (CVVH) or hemodialfiltration (CVVHDF) [1]. In a study of milrinone use in patients with heart failure undergoing CVVH, the steady state concentration and mean half-life was much higher and longer than those previously reported for patients with normal kidney function [2]. High concentration of milrinone has been associated with hypotension and arrhythmia [3]. In contrast, extracorporeal elimination of catecholamines may not be clinically significant since they have a short plasma half-life due to its high non-renal clearance [4]. Critical illness might also affect drug clearance. For example, plasma dopamine clearance has been shown to be lower in critically ill patients and has a large interindividual variation [5]. In an older study, CVVHDF was not found to contribute significantly to the clearance of norepinephrine (NE), epinephrine and dopamine after 4 and 24 hours of treatment [6]. In another small and older study of dopamine clearance with hemodialysis, dopamine clearance was found to be low and may not be clinically important [7].

Vasopressors and inotropes are life-saving medications. Their uses have been incorporated in models that can predict worse outcomes. In one study developing a score to predict mortality within 7 days in patients with sepsis-associated AKI on CKRT, the use of NE was associated with higher odds of mortality [8]. It is likely that this association reflected higher index of severity of illness rather than harm caused by NE in this specific population. In another study of patients who required CKRT, requirement of high dose of vasopressors before CKRT initiation carried a worse prognosis [9]. After mortality, kidney recovery is an important outcome in patients with AKI who require CKRT. Among patients who survived 90 days after CKRT discontinuation, 42% of patients had complete renal recovery [10]. Interventions that may potentially improve renal recovery is important. In a post-hoc analysis of VASST trial, vasopressin compared with NE was associated with a trend to a lower rate of progression of AKI [11]. In the VANISH study, there was less use of kidney replacement therapy (KRT) in the vasopressin group than in the NE group (25.4% for vasopressin vs 35.3% for NE) [12]. This potential differential effects of vasopressin may be due to the reduction of the use of NE [11]. Excessive stimulation of alpha-1 receptor may lead to renal vasoconstriction. Therefore, the use of vasopressor/inotropes may play a role in AKI recovery in patients who receive CKRT. It is important to understand how these drugs are currently being used in patients with AKI on CKRT. In this study, our primary objective was to investigate the impact of using different vasopressors in patients with AKI on CKRT on in-hospital mortality. Our secondary objectives were to investigate the potential impact of different vasopressors on persistent kidney dysfunction and daily fluid balance.

## Material and methods

### Study design

This was a retrospective study. We included patients with AKI who required CKRT at West Virginia University Hospitals between 1/1/2012 and 1/1/2021. We excluded patients if they had end-stage kidney disease, history of kidney transplantation, known pregnancy at admission or who were prisoners. The institutional review board at West Virginia University approved this project and waived the need for informed consent (IRB protocol #2106335895). Patient data was accessed between 6/22/2021 and 2/26/2023. After data collection, the authors did not have access to information that could identify individual patients. STROBE guidelines were followed in reporting the results of this observational study [13].

### Outcomes

The primary outcome was in-hospital mortality. Secondary outcomes included daily fluid balance and persistent kidney dysfunction. Persistent kidney dysfunction was defined as creatinine more than 2-times the baseline value or persistent need for kidney replacement therapy at day 30 after CKRT discontinuation among survivors.

### Statistical analysis

Continuous variables were presented as mean and standard deviation or median and interquartile range (IQR) depending on the variables’ distribution. Frequencies and percentages were used to describe categorical variables. Vasopressor doses were collected when patients started CKRT and throughout their ICU stay. This was collected in increments of 6 hours. The maximum value during that 6-hour period was used as the independent variable. Data on vasopressor use was continued to be collected while patients were in the ICU up until discontinuation of CKRT or day 14. Time-dependent Cox proportional hazard model was used to investigate the outcome of in-hospital mortality. Generalized estimation equation with repeated measure analysis of variance was used to estimate the effect of vasopressors on daily fluid balance. Lastly, the impact of vasopressors on persistent kidney dysfunction was assessed using logistic regression with the mean vasopressor values during the total ICU stay as the independent variable. This is mainly because the methods for examining data with a longitudinal exposure and non-time-varying outcome do not fall in the standard realm of generalized linear mixed models [14]. There is no general consensus on how the information contained in the longitudinal exposure trajectory can be used in a binary regression model [14]. Patients’ charts were manually reviewed to determine the type of shock. This was determined by reviewing the notes of the ICU team. We also explored the effect of norepinephrine equivalent (NEE) dose on outcomes to account for the different vasopressors used [15]. All analysis was done using SAS software 9.4 (SAS Institute Inc., Cary, NC, USA).

## Results

### Patient demographic

There were 641 patients with AKI that required CKRT. In-hospital mortality occurred in 318 (49.6%) patients. Patients who died were more likely to be older (63 vs 57 years), have higher SOFA score (10.6 vs 9), have higher Charlson Comorbidity Index (CCI) (8 vs 7) and have higher NEE dose (0.073 vs 0) mcg/kg/min and were more likely to be mechanically ventilated (80% vs 63%) at CKRT initiation, p<0.05 for all comparisons. Table 1.

**Table 1:** Patient demographics.

	In-hospital mortality (N = 318)	Alive at discharge (N = 323)	P-value
**Age, years [mean (Sd)]**	63 (14)	57 (14)	<0.001
**Weight, Kg [mean (Sd)]**	100 (32)	99 (32)	0.7
**SOFA score on CRRT initiation day [mean (Sd)]**	10.6 (3)	9 (3)	<0.001
**Charlson Comorbidity Index [median (IQR)]**	8 (5;10)	7 (5;9)	0.006
**Female, count (%)**	127 (40%)	135 (42%)	0.7
**Baseline creatinine, mg/dl [mean (Sd)]**	1.4 (0.7)	1.3 (0.7)	0.8
**Mean arterial pressure at CKRT initiation, mmHg [median (IQR)]**	73 (65;81)	78 (69;87)	<0.001
**Lactate, mmol/L [median (IQR)]**	3.5 (1.7; 8.75)	1.8 (1.1;3.7)	<0.001
**Norepinephrine equivalent dose at CKRT initiation, [median (IQR)]** ^**α**^	0.073 (0; 0.23)	0 (0;0.07)	<0.001
**Fluid balance before CKRT initiation, liters [median (IQR)]**	0.32 (0; 2)	0.8 (0; 2.2)	0.1
**Diagnosis of cardiogenic shock, [count (%)]**	103 (32%)	95 (29%)	0.4
**Diagnosis of septic shock, [count (%)]**	258 (81%)	235 (73%)	0.01
**Mechanical Ventilation [count (%)]**	255 (80%)	202 (63%)	<0.001

### Vasopressor use pattern

NE was the most commonly used vasopressor (18% of the time). This was followed by vasopressin (10%), epinephrine (3%) and phenylephrine (2%). Dopamine was used in less than 0.5% of the time and milrinone was not used in our cohort.

Among patients on vasopressin at any given time (10%), patients were on at least one other vasopressor in 88% of the time. Among patients on NE at any given time (18%), patients were on at least one other vasopressor in 48% of the time. Table 2.

**Table 2:** Vasopressor use pattern.

Vasopressor	Time on each Vasopressor (%)	Time on ≥1 Other Vasopressor (%)	Time on ≥2 Other Vasopressors (%)	Time on Vasopressor Alone (%)	Time Not on Vasopressor (%)
Vasopressin	10	88	38	11	90
Epinephrine	3	73	50	27	97
Phenylephrine	2	70	50	30	98
Norepinephrine	18	48	21	52	82

Patients were classified according to the type of shock. There were 353 (55%) patients who had septic shock only, 58 (9%) as cardiogenic shock only, 140 (22%) patients as having both types during their ICU stay and 90 (14%) patients as neither septic nor cardiogenic shock. NE was used the most when both types of shock were documented (21%) rather than either one alone (septic shock only 19% vs cardiogenic shock only 11%). Vasopressin was used the most when both types of shock were present (16%) vs when cardiogenic shock only (11%) or septic shock only (9%), p<0.001. Similarly, epinephrine was used the most when both types of shock were present compared to when either alone was documented (both 15%, cardiogenic only 10.5% and septic shock only 3%). Phenylephrine was rarely used in cardiogenic shock only (0.3%), 1.5% when both types present and 3% when septic shock only were present, p<0.001 for all.

### In-hospital mortality and persistent kidney dysfunction

In multivariable Cox proportional hazard model, increasing doses of norepinephrine [HR 4.4 (95% CI: 2.3–7, p<0.001)] and vasopressin [HR 2.6 (95% CI: 1.9–3.2, p = 0.01)] during CKRT were associated with in-hospital mortality per 0.02 increase in the dose. Dobutamine, epinephrine and phenylephrine were not associated with in-hospital mortality. Dopamine was used in less than 0.5% of the time and was not analyzed. Baseline vasopressor values measured at CKRT initiation were not associated with in-hospital mortality. The model was adjusted for mean arterial pressure, vasopressor doses and fluid balance prior to CKRT initiation, time-dependent daily fluid balance and vasopressor doses, SOFA score, septic shock, age, sex, CCI, mechanical ventilation and baseline lactate. Table 3.

**Table 3:** Factors associated with in-hospital mortality.

Variable	adjusted HR (95% CI)	p-value
Norepinephrine (per 0.02 mcg/kg/min)	4.4 (2.3–7)	<0.001
Vasopressin (per 0.02 unit/min)	2.6 (1.9–3.2)	0.01
Epinephrine (per 0.02 mcg/kg/min)	1.1 (0.9–2)	0.9
Phenylephrine (per 1 mcg/kg/min)	1.1 (0.95–1.2)	0.2
Dobutamine (per 1 mcg/kg/min)	1 (0.9–1.1)	0.8
Daily fluid balance (per 1 L)	1.13 (1.06–1.2)	<0.001
Lactate (per 1 mmol/L)	1.06 (1.03–1.08)	<0.001
Age (per 1 year)	1.02 (1.01–1.03)	<0.001
SOFA score (per 1 unit)	1.09 (1.05–1.13)	<0.001
Septic Shock	0.95 (0.7–1.3)	0.7
Female sex	1.07 (0.8–1.3)	0.5
CCI	1.01 (0.9–1.05)	0.7
Mechanical ventilation	1.3 (0.98–1.8)	0.07

Model adjusted for: mean arterial pressure, vasopressor doses and fluid balance prior to CKRT initiation, time-dependent daily fluid balance and vasopressor doses, SOFA score, septic shock, age, sex, CCI, mechanical ventilation and baseline lactate.

There were differences between association of different vasopressors with mortality when stratifying the analysis by the type of shock. In patients who were labeled as having both cardiogenic and septic shock during their ICU stay, only the use of epinephrine was associated with in-hospital mortality HR 1.1 (95% CI: 1.01–1.23, p = 0.04). In patients with cardiogenic shock only, no vasopressor was associated with increased mortality. In patients with septic shock only, the use of NE was associated with increased mortality HR 4.9 (95% CI: 2.4–9, p<0.001).

Among survivors at day 30, mean doses of vasopressors during ICU stay were not associated with persistent kidney dysfunction. There was also no specific combination of vasopressors that was associated with persistent kidney dysfunction.

### Fluid balance

All vasopressors were associated with positive fluid balance. The coefficients varied with epinephrine having the highest coefficient 4.3 L and phenylephrine having the lowest coefficient 0.6 L. Table 4. The model was adjusted for the same variables considered in the mortality outcome.

**Table 4:** Association with time-dependent fluid balance.

Variable	adjusted coefficient (95% CI)	p-value
Norepinephrine (per 0.02 mcg/kg/min)	2.5 (2–3)	<0.001
Vasopressin (per 0.02 unit/min)	2.4 (1.2–3.5)	<0.001
Epinephrine (per 0.02 mcg/kg/min)	4.3 (2.4–6.4)	<0.001
Phenylephrine (per 1 mcg/kg/min)	0.6 (0.45–0.73)	<0.001
Dobutamine (per 1 mcg/kg/min)	-0.01 (-0.06–0.04)	0.7
Lactate (per 1 mmol/L)	0.04 (0.02–0.06)	<0.001
Age (per 1 year)	0.007 (0.001–0.012)	0.01

Model adjusted for: mean arterial pressure, vasopressor doses and fluid balance prior to CKRT initiation, time-dependent daily fluid balance and vasopressor doses, SOFA score, septic shock, age, sex, CCI, mechanical ventilation and baseline lactate.

Considering the pharmaceutical preparation of the specific vasopressor and the average time patients were on specific vasopressor, it was expected that patients on NE or vasopressin to receive the highest volume of carrying fluid, S1 Table.

## Discussion

In this study, use of NE and vasopressin were associated with worse in-hospital mortality among patients with AKI on CKRT. All vasopressors were associated with positive fluid balance. None of the vasopressors were associated with persistent kidney dysfunction among survivors at day 30. The results of this study highlight the varying pattern of utilization of vasopressors among patients with AKI on CKRT.

The use of vasopressors in patients on CKRT has not been well-studied. One study investigated the effect of varying hemofiltration doses (high vs low) on the effect of NE requirement [16]. This was mostly testing the effect of potential cytokine removal and showed decreased NE use with high dose hemofiltration. Another study showed worse outcomes in patients with AKI who required NE prior to kidney replacement therapy; however, the modality of KRT was chosen by the nephrologist [17]. While the authors used inotropic equivalent to account for the different groups (NE users vs non-NE users), the use of other vasopressors was not mentioned in details. In a study evaluating the use of high-dose of vasopressor used before the initiation of CKRT, dopamine dose of ≥20 mcg/kg/min and NE dose of ≥0.3 mcg/kg/min were significantly associated with mortality [9]. It is important to consider the time patients were on vasopressors rather than assessing the association with mortality at one particular time. In our study, baseline vasopressor doses were not associated with mortality; however, when we considered vasopressors as time-dependent variables, NE and vasopressin were found to be associated with in-hospital mortality.

The pharmacokinetics of vasopressors might be affected by kidney function. Milrinone is highly protein bound and is predominately eliminated by renal excretion with approximately 80% eliminated as unchanged drug in the urine [3]. It was found that higher dose of milrinone were independently associated with development of new-onset atrial fibrillation in patients with AKI on CKRT [18]. Conversely, dobutamine was not associated with increased rate of cardiac arrhythmias in patients with end-stage kidney disease on hemodialysis [19]. In patients with AKI on CKRT, one cannot conclude if certain vasopressor or inotrope may be associated with worse outcomes. This is due to differences in study design (dose of a medication at a one particular time or prior to starting CKRT), heterogenous patient population (AKI vs end-stage kidney disease) and different modalities (hemodialysis vs CKRT). AKI is considered a heterogenous syndrome and because of that there is lack of effective therapies. Nonetheless, in a study that sought to identify patients with AKI phenotypes, two classes were identified that shared similar clinical and physiological characteristics within their class [20]. Using the VASST cohort, patients identified as belonging to AKI subphenotype I benefited from vasopressin compared to NE whereas there was no effect of vasopressin in subpheotype II [20]. Other studies have shown similar association between the use of specific vasopressor in certain types of shock. In a systematic review, using network meta-analysis NE plus dobutamine was associated with a lower risk of 28-day mortality in septic shock patients than other vasoactive medications [21]. The use of dopamine was associated with a higher risk of 28-day mortality due to septic shock than NE, terlipressin, and vasopressin [21]. In our study, among patients with septic shock only, NE was the only vasopressor to be associated with worse mortality. While this could be attributed to its common use in this population, this is the first study that investigated the use of different vasopressors while considering the duration of exposure in patients receiving CKRT. The absence of association between the rest of the vasopressors with mortality could be due to their less common use.

While it is important to maintain end-organ perfusion, increased doses of NE may come at the expense of compromising organ-specific oxygen consumption and perfusion [22]. In animal models of sepsis associated AKI, resuscitation with NE lead to decreased medullary perfusion and partial pressure of oxygen. These developed before AKI was detected suggesting that they may be critical mediators in the initiation and progression of septic AKI [23]. In a post-hoc analysis of the VASST trial, use of vasopressin was associated with a lower rate of progression of AKI when compared to NE [11]. Similarly, in patients with vasoplegic shock after cardiac surgery, rates of kidney injury occurred more in the NE group vs the vasopressin group (35.8% versus 10.3%) [24]. Also, higher urine volumes and lower rate of KRT were found with early addition of vasopressin to NE in septic shock [12]. Nonetheless, there remains no high-quality evidence of superiority of one vasopressor over the other in AKI recovery. In patients with septic shock, phenylephrine did not confer kidney benefits when compared to NE with urine output and creatinine clearance being similar between the two groups [25]. In adult patients undergoing major noncardiac surgery under general anesthesia, the rates of AKI were similar between patients using NE versus phenylephrine as the first-line vasopressor [26]. Although NE was associated with progression of AKI in patients with severe sepsis, this was the maximum dose received within the first 5 days of ICU. The relationship was no longer statistically significant in adjusted model [27]. Among patients with septic shock there was no difference in KRT between NE and dopamine use [28]. In our study, there was no difference in persistent kidney dysfunction at day 30 among survivors between different vasopressors.

Another adverse effect we considered in this study was fluid overload. We expected that one vasopressor might be more likely to lead to more positive fluid balance by nature of the pharmaceutical preparation and dilution. However, all vasopressors were associated with higher positive fluid balance. This is a similar finding to the literature. In adult patients undergoing major noncardiac surgery under general anesthesia, there was no difference in fluid balance between NE and phenylephrine use [26]. A similar result was found among patients with septic shock when comparing patients on NE vs phenylephrine as the initial drug [29].

Our study had some limitations. First, while we accounted for most of the potential confounders, it remains a retrospective study and is subject to unmeasured biases. Second, this was a single center study and thus the findings may not be generalizable. This would depend on each center’s protocols for various vasopressor use and titration. However, we acknowledge some strengths in this study. We showed that contrary to the general risk predictions that use baseline values of vasopressor, the use of vasopressor doses during CKRT is more valuable. Baseline values do not capture patient’s risk of mortality. We add to the literature that no specific vasopressor was associated with persistent kidney dysfunction at day 30.

In conclusion, the use of NE and vasopressin were associated with higher in-hospital mortality in patients with AKI on CKRT. This may be driven by their common use in patients with high degree of severity of illness. Future studies are important to inform clinicians whether the use of specific vasopressor in patients on CKRT would alter their outcome.

## Supporting information

S1 Table Vasopressor dosing and carrying fluid volume. (DOCX)
