# Supplementary material for: Use of vasopressors in patients with acute kidney injury on continuous kidney replacement therapy
Source: PLoS One. 2024 Dec 19;19(12):e0315643. doi: 10.1371/journal.pone.0315643 (PMC11658609; doi:10.1371/journal.pone.0315643)
Supplement: S1 Table — (DOCX) [file pone.0315643.s001.docx]

**Supplementary Table 1.** Vasopressor dosing and carrying fluid volume

| Vasopressor | Average Dose received | Weight (Kg) | Average time  (minutes) | Average dose  (in mcg or units) | Dose in mcg/ml (or units/ml) | Expected total volume received on average (ml) |
| --- | --- | --- | --- | --- | --- | --- |
| NE (mcg/kg/min) | 0.12 | 75 | 3720 | 33480 | 64 | 523.125 |
| Epinephrine (mcg/kg/min) | 0.085 | 75 | 3768 | 24021 | 100 | 240.21 |
| Phenylephrine (mcg/kg/min) | 2.6 | 75 | 2472 | 482040 | 20000 | 24.102 |
| Vasopressin (units/min) | 0.04 | NA | 3984 | 159.36 | 0.2 | 796.8 |

The average time denotes minutes patients were on specific vasopressor.

Doses in mcg/ml or units/ml were calculated based on the preparation of the specific vasopressor below: Norepinephrine: 16mg in 250 ml normal saline. Epinephrine: 25 mg in 250 ml normal saline. Phenylephrine: 5000 mg in 250 ml normal saline. Vasopressin: 20 units in 100 ml normal saline.
